# Prevalence and etiologies of pulmonary hypertension in Africa: a systematic review and meta-analysis

**DOI:** 10.1186/s12890-017-0549-5

**Published:** 2017-12-08

**Authors:** Jean Joel Bigna, Jean Jacques Noubiap, Jobert Richie Nansseu, Leopold Ndemnge Aminde

**Affiliations:** 1 Department of Epidemiology and Public Health, Centre Pasteur of Cameroon, Yaoundé, Cameroon; 2 Faculty of Medicine, University of Paris Sud XI, Le Kremlin Bicêtre, France; 3 Department of Medicine, Groote Schuur Hospital and University of Cape Town, Cape Town, South Africa; 4 Department of Public Health, Faculty of Medicine and Biomedical Sciences, University of Yaoundé 1, Yaoundé, Cameroon; 5 Sickle Cell Disease Unit, Mother and Child Centre of the Chantal Biya Foundation, Yaoundé, Cameroon; 6 Department of Disease, Epidemics and Pandemics Control, Ministry of Public Health, Yaoundé, Cameroon; 7 Clinical Research Education, Networking and Consultancy (CRENC), Douala, Cameroon; 8 School of Public Health, Faculty of Medicine & Biomedical Sciences, University of Queensland, Brisbane, Australia

**Keywords:** Pulmonary hypertension, Pulmonary arterial hypertension, Africa, Systematic review, Meta-analysis, Epidemiology

## Abstract

**Background:**

Despite the recent increasing worldwide attention towards pulmonary hypertension (PH), its epidemiology remains poorly described in Africa. Accordingly, we performed a systematic review and meta-analysis of PH prevalence, incidence and etiologies in Africa.

**Methods:**

We searched PubMed, EMBASE, African Journals Online, and Africa Index Medicus. Published observational studies until September 20, 2017, including adult participants residing in Africa were considered. Two review authors independently selected studies, assessed included studies for methodological quality, and extracted data. A random-effects model was used for meta-analysis. Heterogeneity was evaluated by the *χ* ^2^ test on Cochrane’s Q statistic which is quantified by I^2^ values. Using Newcastle-Ottawa Scale, we considered a score of 0–4, 5–7, and 8–10 as indicative of high, moderate, and low risk of bias in included studies, respectively.

**Results:**

Of 1611 entries, 25 studies were retained. Twelve (48%), seven (28%), and six (24%) papers had respectively a low, moderate and high risk of bias. The prevalence of PH widely varied across different populations: 9.8% (95% confidence interval: 3.2–19.3; I^2^ = 99.4%; 6 studies) in 11,163 people presenting with cardiac complaints; 10.6% (4.3–19.1; I^2^ = 90.3%; 4 studies) in 937 HIV-infected people; 32.9% (17.6–50.4; I^2^ = 97.2%; 3 studies) in 2077 patients with heart failure; 23.2% (15.2–32.2; I^2^ = 59.4%; 3 studies) in 248 patients on hemodialysis; 12.9% (11.8–14.0; I^2^ = 79.7%; 2 studies) in 3750 patients with rheumatic heart disease; 36.9% (29.7–44.3; I^2^ = 79.7; 2 studies) in 79 patients with sickle cell disease; 62.7% (49.0–74.7; 1 study) in 51 patients with chronic obstructive pulmonary disease; 25.4% (16.3–37.3; 1 study) in 63 patients with systemic lupus erythematous; 68.7% (62.8–74.1; 1 study) in 259 patients with cardiac surgery; and 7.4% (4.6–11.9; 1 study) in 202 patients with systemic sclerosis. No study reported PH incidence. From one international study (*n* = 209), PH etiologies were: left heart disease (68.9%), pulmonary arterial hypertension (15.8%), lung disease and/or hypoxia (12.0%), chronic thromboembolic PH (1.9%) and unclear/multifactorial PH (15.8%).

**Conclusion:**

The prevalence of PH is relatively high in some populations in Africa, perhaps mainly driven by left heart diseases, highlighting the need for context-specific interventions.

**Electronic supplementary material:**

The online version of this article (10.1186/s12890-017-0549-5) contains supplementary material, which is available to authorized users.

## Background

Pulmonary hypertension (PH) is a hemodynamic and pathophysiological condition characterized by abnormally elevated pressures in the pulmonary vasculature. It is defined by a mean pulmonary arterial pressure ≥ 25 mmHg at rest by right heart catheterization [1]. PH can be caused by an increase in pulmonary blood flow, pulmonary vascular resistance, pulmonary venous pressure or a combination of these factors. It is classified into five main groups of causes, each group sharing similar pathophysiological features: pulmonary arterial hypertension (group 1), PH due to left heart disease (group 2), PH due to lung disease and/or hypoxia (group 3), chronic thromboembolic PH (group 4) and PH with unclear/multifactorial mechanisms (group 5) [2].

Irrespective of the cause, PH is associated with debilitating symptoms and reduced life expectancy. Late diagnosis and ineffective treatment are the main drivers of its poor survival [3, 4]. For instance, pulmonary arterial hypertension (PAH) has a median survival of 2.8 years if the patient is not treated with improved treatment regimens [5]. Following the introduction of PAH improved treatment regimens, the prognosis of PAH has improved considerably, with 1-, 3-, and 5-year survival rates of 85%, 68%, and 57%, respectively [6]. Early diagnosis and appropriate treatment of PH are therefore paramount in order to improve its outcomes. Many known risk factors for PH are hyperendemic in Africa, including human immunodeficiency virus (HIV) infection, acquired immunodeficiency syndrome (AIDS), rheumatic heart disease, chronic hepatitis B and C, hereditary hemoglobinopathies, tuberculosis, asthma, and schistosomiasis [7–16]. Moreover, left heart disease which is increasingly prevalent in African populations has been shown to be an important cause of PH across the continent [14, 15, 17]. Therefore, the epidemiological and clinical profiles of diseases differ from country to country and from continent to continent. It is why precise knowledge of region-specific epidemiology of PH is crucial to implement effective preventive strategies and for contextualized clinical guidelines.

Despite the point made above and the potential risk of highly prevalent PH, the epidemiology of PH remains poorly described in Africa. In fact, there is a lack of studies on PH prevalence from this continent [16]. High prevalence of PH risk factors, specific genetic background and lifestyles, poor access to health care suggest that the epidemiology of PH in Africa may be unique.

Herein, we present the first systematic review and meta-analysis which synthesizes the current knowledge on the epidemiology of PH in Africa. Our objective was to determine the prevalence, the incidence and the etiologies of PH among people residing in Africa. The ultimate goal of this review is to provide evidence which could guide contextualized policies for the prevention and treatment of PH in Africa, and underpin further research.

## Methods

The MOOSE guidelines served as the template for reporting the present review [18]. This review was registered in the PROSPERO International Prospective Register of systematic reviews, registration number CRD42016049351 and its protocol was published [19].

### Criteria for considering studies for the review

Published observational studies until September 20, 2017, including adult (18 years or older) participants residing in Africa were considered. Studies were also considered if the diagnosis of PH was based on right heart catheterization with a mean pulmonary arterial hypertension ≥25 mmHg or Doppler echocardiography examination with pulmonary arterial systolic pressure > 35 mmHg [1].

### Search strategy for identifying relevant studies

An expert librarian performed a search of PubMed/MEDLINE, Excerpta Medica Database, African Journals Online and African Index Medicus without any language restriction. The search strategy included the following terms: ‘Africa’, ‘pulmonary hypertension’, and ‘pulmonary arterial hypertension’. Individual country names for the 54 African countries were also used as additional key search terms to identify more abstracts on the subject. References of all relevant original and review articles were scrutinized for additional potential data sources. The main search strategy conducted in PubMed is available in the review protocol [19].

### Study selection

Two review authors (JJB and JJN) independently screened abstracts and then full texts. They consensually retained all studies to be included in the review, and disagreements were solved by arbitration of a third review author (JRN).

### Assessment of the methodological quality of included studies

The Newcastle-Ottawa Scale was used to evaluate the methodological quality of studies included in this review [20]. There is no validation study that provides a cut-off score for rating low-quality studies. We considered 0–4, 5–7, and 8–10 stars as indicative of high, moderate, and low risk of bias, respectively. Two investigators (JJB and JJN) independently assessed study quality, with disagreements resolved by consensus.

### Data extraction and management

Two review authors (JJB and JJN) independently extracted data including: first author name, year of publication, year of participants’ inclusion, country, study design, setting, sample size, age distribution, proportion of males, diagnostic criteria for PH, number of cases of PH, and etiologies of PH as classified by international guidelines [2]. Authors were contacted at least twice to request relevant missing information. A World Health Organization Afro sub-region was assigned to each study based on the country of recruitment.

### Data synthesis and analysis

A meta-analysis was conducted for data obtained from studies in the same population (patients with chronic obstructive pulmonary disease, with heart failure, on hemodialysis, presenting with cardiac complaints, with HIV infection, with rheumatic heart disease, with sickle cell disease, with systemic lupus erythematous, with cardiac surgery, and with systemic sclerosis). Standard errors for the study-specific estimates were determined from the point estimate and the appropriate denominators. Then, the study-specific estimates were pooled through a random-effects meta-analysis model, to obtain an overall summary estimate of the prevalence across studies, after stabilizing the variance of individual studies using the Freeman-Tukey double arc-sine transformation [21]. Heterogeneity was evaluated by the *χ*2 test on Cochrane’s Q statistic which is quantified by I^2^ values [22], assuming that I^2^ values of 25%, 50% and 75% respectively represent low, medium and high heterogeneity [23]. Inter-rater agreement for study inclusion was assessed using Cohen’s kappa (κ) coefficient [24]. Egger’s test served to detect publication bias [25]. A *p* value <0.1 was considered indicative of statistically significant publication bias. Data were analyzed using Stata software (Stata Corp. 2013. *Stata Statistical Software: Release 13*. College Station, TX: StataCorp LP). We conducted a narrative synthesis in the case of limited data for a meta-analysis.

## Results

### The review process

**Fig. 1:**
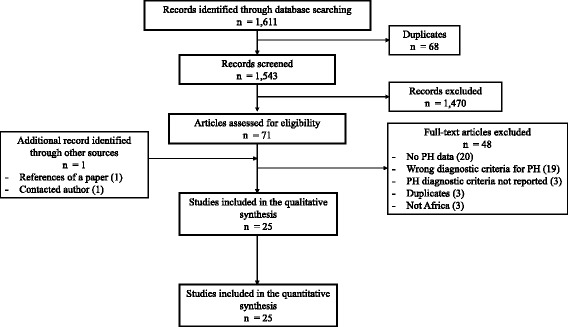
The review process

Initially, a total of 1611 records were identified. After elimination of duplicates, 1543 records were retained. Titles and abstracts were screened and 1470 records were found irrelevant and excluded. Agreement between review authors on abstract selection was moderate (κ = 0.69). Full-texts of the remaining 73 papers were scrutinized for eligibility, among which 48 were excluded (Fig. 1). Overall, 25 papers were found eligible and were included in the data synthesis; 24 papers reported the prevalence of PH [26–49], while one other paper reported PH etiologies [17]. The inter-rater agreement for final study inclusion between review authors was high (κ = 0.93).

### Methodological quality and characteristics of included studies

The characteristics of each included study are presented in Additional file 1. Twelve (48%), seven (28%), and six (24%) papers had respectively a low, moderate and high risk of bias in their methodological quality [See Additional file 2]; all were cross sectional studies. The diagnosis of PH was performed using right heart catheterization in only one study while the rest used echocardiography. The studies included were published from 2003 to 2017 and reported surveys conducted from 2004 to 2015. Eight studies were from Northern, five from Central, four from Eastern, three from Southern, and three from Western Africa. Two studies included data from different countries. Nineteen studies prospectively collected data and the others, retrospectively. Twenty-four studies included participants from both rural and urban areas and one from urban areas only. Participants were randomly selected only in two studies. Twenty-three studies were hospital-based and the others were community-based. Mean/median ages reported in 18 studies varied from 27 to 64 years; the age ranged from 17 to 98 years. The male proportion reported in 17 studies varied from 5 to 100%.

### Prevalence and incidence of pulmonary hypertension in Africa

**Fig. 2:**
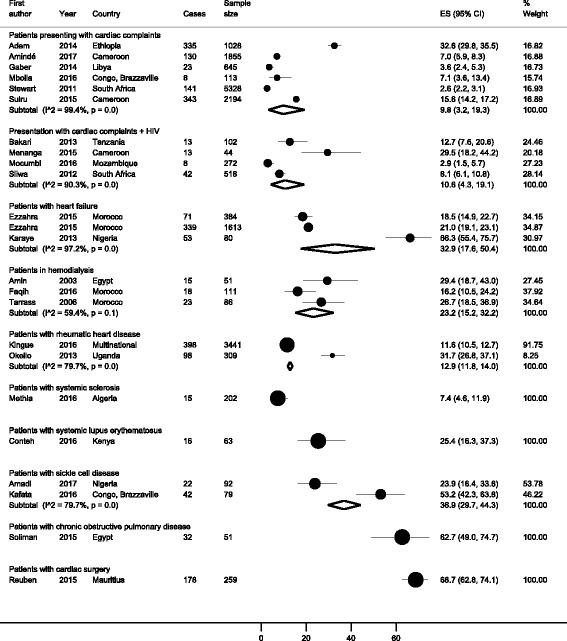
Meta-analysis results for prevalence of pulmonary hypertension in Africa

Figure 2 presents the prevalence of PH in specific groups. Among patients presenting with cardiac complaints, the prevalence was 9.8%, 95% confidence interval (CI): 3.2–19.3, I^2^ = 99.4%; in 11,163 participants from six studies conducted in Cameroon, Congo Brazzaville, Ethiopia, Libya and South Africa (p Egger = 0.478) [26, 33, 37, 45, 46, 49]. Among HIV-infected patients presenting with cardiac complaints, the prevalence was 10.6% (95%CI: 4.3–19.1; I^2^ = 99.6%) in a pooled sample of 937 patients from four studies conducted in different countries including Cameroon, Mozambique, South Africa and Tanzania (p Egger = 0.351) [28, 38, 40, 43]. A prevalence of 32.9% (95%CI: 17.6–50.4; I^2^ = 97.2%) was found in a pooled sample of 2077 patients with heart failure from three studies conducted in Morocco and Nigeria (p Egger = 0.445) [30, 31, 35]. A prevalence of 23.2% (95%CI: 15.2–32.2; I^2^ = 59.4%) was found in a pooled sample of 248 patients undergoing hemodialysis from three studies conducted in Egypt and Morocco (p Egger = 0.376) [27, 32, 47]. A prevalence of 12.9% (95%CI: 11.8–14.0; I^2^ = 96.8%) was found in a pooled sample of 3750 patients with rheumatic heart disease from two studies, one from Uganda and one international (Cameroon, Ivory Coast, Guinea-Conakry, Mali, Nigeria, Senegal, and Togo) [36, 41]. A prevalence of 36.9% (95%CI 29.7–44.3; I^2^ = 79.7) was found in a pooled sample of 171 people with sickle cell disease from two studies conducted in Congo Brazzaville and Nigeria [34, 48].

In one study conducted in Egypt, the prevalence of PH was 62.7% (95%CI: 49.0–74.7) among 51 patients with chronic obstructive pulmonary disease [44]. One other study reported a prevalence of 25.4% (95%CI: 16.3–37.3) in 63 patients with systemic lupus erythematous in Kenya [29]. In one study conducted in Mauritius, the prevalence of PH was 68.7% (95%CI: 62.8–74.1) among 259 patients with cardiac surgery [42]. One study reported a prevalence of 7.4% (95%CI: 4.6–11.9) in 202 patients with systemic sclerosis in Algeria [39]. No study reported the incidence of PH.

### Etiologies of pulmonary hypertension in Africa

Only one study, the Pan African Pulmonary hypertension Cohort (PAPUCO) study, reported the etiologies of PH as classified by Simmoneau and colleagues [17]. This study included 209 subjects with a median age of 48 years; the study population comprised 41% of males and 35% of HIV-infected patients recruited in Cardiology units in Cameroon, Mozambique, Nigeria and South Africa. The etiologies were distributed as follows: 144 (68.9%) presented with PH due to left heart disease, 33 (15.8%) with PAH, 25 (12.0%) with PH due to lung disease and/or hypoxia, 4 (1.9%) with chronic thromboembolic PH and 33 (15.8%) with unclear/multifactorial PH.

## Discussion

Although, we have neither found studies reporting the prevalence of PH in the general population nor those reporting the incidence of PH in Africa, available studies showed that the prevalence of PH may vary widely across specific populations: from 7.4% in patients with systemic sclerosis to 68.7% in patients with cardiac surgery. The main driver of PH among patients presenting with cardiac complaints may be left-sided heart disease, which seems to represent two-thirds of PH etiologies in Africa.

Among patients presenting with cardiac complaints, the prevalence of PH was 9.8% which is in the 10–20% range reported in the US general population [50]. In more specific populations, we found a high prevalence of PH in patients with well-known causes of PH including group 1: systemic lupus erythematous (25.4%), systemic sclerosis (7.4%), and HIV infection (10.6%). The prevalence of PH in patients with systemic sclerosis in this review was close to the prevalence reported in developed countries, albeit measured with right heart catheterization [51]. Concerning HIV-infected people, our prevalence estimate is almost on par with a previous review in the same population in Africa [9]. The prevalence of PH among HIV-infected individuals in Africa remains higher than the 0.5% reported from developed countries [52–54]. This can be explained by several reasons including low access and retention in care of HIV-infected people in Africa leading to late diagnosis and management of HIV disease (antiretroviral therapy initiation) [9, 55–58]. Further, we noticed a higher prevalence of PH among Africans with systemic lupus erythematous compared to the prevalence reported in developed countries (0.5–17.5%) [51]. Due to fact, that many known risk factors for PH are hyperendemic in Africa [7–16], patients with systemic lupus erythematous or HIV residing in Africa probably had other comorbid conditions favoring the occurrence of PH.

Group 2 causes included: rheumatic heart disease (12.9%), heart failure (32.9%), and cardiac surgery (68.7%). Similarly, a recent global systematic review by Dzudie and colleagues reported a prevalence of PH varying from 22 to 83.3% in adults with left heart disease [17, 59]. Indeed, left heart diseases represent the most common causes of PH [60–62]. Actually, it has been observed that even after surgery for rheumatic heart disease complicated by PH, some residual PH is seen to persist [63].

For group 3 causes of PH, we found a single study from Egypt, which reported a prevalence of 62.7% among those with chronic obstructive pulmonary disease. This is higher than findings from developed countries among patients with end-stage chronic obstructive pulmonary disease (48.7%) [64]. For group 5 causes of PH, the 23.2% prevalence of PH in patients with hemodialysis in our review falls within the range reported from other settings (18.8–68.8%) [65]. In contrast to developed countries where the reviews on the prevalence of PH in sickle cell disease individuals reported a range between 6 and 11% [66, 67], we found that 36.9% of patients with sickle cell disease had PH. We found no study conducted among patients with group 4 PH (chronic thromboembolic PH). While this might look like a rarity of chronic thromboembolic PH in Africa, the limited availability (and affordability) of the complex diagnostic tools investigating lung function required for accurate diagnosis of this form of PH, likely explains the dearth in studies and potentially underdiagnoses.

Overarchingly, one can note that the prevalence of PH in different specific populations in Africa is either similar to, or in the majority of situations higher than that reported in other non-African settings. This is potentially explained by the presence of many known endemic risk factors for PH in Africa (some not fully explored) [7–16]. We found just one study classifying PH according to current guidelines. Despite the dearth in studies, available evidence suggests that the main class of PH in Africa is the one due to left heart diseases which is consistent with prior literature [62, 68–70].

About half of studies included in this review had a low risk of bias in their methodological quality. We observed no publication bias, suggesting that future research endeavors are likely to have no important impact on the confidence in our estimates. A key exclusion criterion for studies during the review process was wrong diagnostic criteria for PH (i.e. a cut-off different from 35 mmHg for pulmonary arterial systolic pressure on echocardiography). Indeed, some studies used a cut-off between 20 and 30 mmHg [71–79] and others between 37 and 50 mmHg [80–85] on echocardiography. Most studies in this review used Doppler echography for the diagnosis of PH, and were hospital-based; moreover, PH was mainly symptom-driven (vs. general population). In this respect, interpretation of our findings should be done with caution.

Further studies are needed to investigate PH etiologies in Africa. Clinicians and researchers are invited to comply with updated guidelines for accurate diagnosis and classification of PH, as recommended by international guidelines [86]. While the PAPUCO study [17] was a commendable effort describing the natural history of PH in Africa, there is still need for further studies and collaborations to expand these strides and provide more robust evidence for Africa. However, the high prevalence of PH found in this review, albeit in specific populations, underscores the need for clinicians, policy makers, researchers and stakeholders to direct more attention towards PH in a bid to improve detection and management of the disease throughout the continent. What we need in Africa are studies that can develop some clinical screening methods that can help to identify almost all PH cases in community. Indeed, early diagnosis and timely access to care can improve the clinical course of the disease and potentially reduce its burden. The greatest difficulty is to know how to ensure that everyone at risk to develop PH has access to its eventual diagnosis in a weak healthcare system and in a context of constrained resources. We also hope that all these efforts will bring to define local and adapted guidelines for the diagnosis and management of PH in Africa, taking into account the specific patterns of the disease across the continent.

This review had some limitations. First, although we searched several databases, this review is based on a limited number of original studies published in specific populations. Second, the limited number of studies included in the review did not permit us to perform meaningful subgroup analyses to determine sources of heterogeneity. Third, not all African sub-regions were represented in all the specific populations, making the results of this study particular in terms of generalizability to the entire continent. Nonetheless, this is the first comprehensive review on the prevalence, incidence and etiologies of PH in Africa; it gives a clear overview of the burden of PH in Africa and crucial gaps to fill in future research.

## Conclusions

This review suggests that the prevalence of PH is relatively high in some populations in the African context. These high prevalence estimates seem to be driven by left heart diseases. To tackle this high disease burden, policy makers and healthcare providers must be aware of this reality and invest in interventions towards improving prevention, detection and management of PH in Africa. Community-based studies are required to better characterize the epidemiology and natural history of PH in Africa.

## Additional files

Additional file 1: Individual characteristics of included studies. (PDF 634 kb)

Additional file 2: Risk of bias in individual studies. (PDF 287 kb)

## Abbreviations

PAH: Pulmonary arterial hypertension
PAPUCO: Pan African Pulmonary hypertension Cohort study
PH: Pulmonary hypertension
