# Supplementary material for: Prevalence and etiologies of pulmonary hypertension in Africa: a systematic review and meta-analysis
Source: BMC Pulm Med. 2017 Dec 8;17:183. doi: 10.1186/s12890-017-0549-5 (PMC5723068; doi:10.1186/s12890-017-0549-5)
Supplement: Supplementary file 1 — Individual characteristics of included studies. (PDF 634 kb) [file 12890_2017_549_MOESM1_ESM.pdf]

Supplemental file 1. Characteristics of included studies

First author	Year	Period of inclusion	Countries	Region	Timing of data collection	Study Design	Selection	Area	Setting	Sample	Cases	Prevalence of PH	Etiologies of PH	Mean age, years	Median age, years	Min Age, years	Max Age, years	Male %	Diagnostic tool	Population
Adem	2014	2011-2013	Ethiopia	Eastern	Retrospective	Cross sectional	Not random selection	Urban and Rural	Hospital-based	1028	335	32.6%	NR	51	52	20	98	48	Echocardiography	Patients presenting with cardiac complaints
Amadi	2014	2012	Nigeria	Western	Prospective	Cross sectional	Not random selection	Urban and Rural	Hospital-based	92	22	23.9	NR	25.4		18	41	4537	Echocardiography	Patients with sickle cell disease
Amin	2003		Egypt	Northern	Prospective	Cross sectional	Not random selection	Urban and Rural	Hospital-based	51	15	29.4%	NR	43	NR	NR	NR	55	Echocardiography	Patients in hemodialysis
Bakari	2013	2009-2010	Tanzania	Eastern	Prospective	Cross sectional	Not random selection	Urban and Rural	Hospital-based	102	13	12.7%	NR	42	NR	18	72	31	Echocardiography	Presentation with cardiac complaints + HIV
Conteh	2016	2015	Kenya	Eastern	Prospective	Cross sectional	Not random selection	Urban and Rural	Hospital-based	63	16	25.4%	NR	38	NR	17	59	5	Echocardiography	Patients with systemic lupus erythematosus
Ezzahra	2015		Morocco	Northern	Prospective	Cross sectional	Not random selection	Urban and Rural	Hospital-based	384	71	18.5%	NR	64	NR	NR	NR	60	Echocardiography	Patients with heart failure
Ezzahra	2015		Morocco	Northern	Retrospective	Cross sectional	Not random selection	Urban and Rural	Hospital-based	1613	339	21.0%	NR	NR	NR	NR	NR	NR	Echocardiography	Patients with heart failure
Faqih	2016	2014	Morocco	Northern	Prospective	Cross sectional	Not random selection	Urban and Rural	Hospital-based	111	18	16.2%	NR	44	NR	NR	NR	49	Echocardiography	Patients in hemodialysis
Gaber	2014	2010-2013	Libya	Northern	Retrospective	Cross sectional	Not random selection	Urban and Rural	Hospital-based	645	23	3.6%	NR	NR	NR	NR	NR	NR	Echocardiography	Patients presenting with cardiac complaints
Kafata	2016		Congo, Brazzaville	Central	Prospective	Cross sectional	Not random selection	Urban and Rural	Hospital-based	79	42	53.2%	NR	27	NR	NR	NR	40	Echocardiography	Patients with sickle cell disease
Karaye	2013		Nigeria	Western	Prospective	Cross sectional	Not random selection	Urban and Rural	Hospital-based	80	53	66.3%	NR	46	NR	NR	NR	39	Echocardiography	Patients with heart failure
Kingue	2016	2004-2008	Cameroon, Ivoiry Coast, Guinea, Mali, Nigeria, Senegal, Togo	Multinational	Prospective	Cross sectional	Not random selection	Urban and Rural	Hospital-based	3441	398	11.6%	NR	31	NR	NR	NR	40	Echocardiography	Patients with rheumatic heart disease
Mbolla	2016	2008-2012	Congo, Brazzaville	Central	Prospective	Cross sectional	Not random selection	Urban and Rural	Hospital-based	113	8	7.1%	NR	NR	NR	NR	NR	34	Echocardiography	Patients presenting with cardiac complaints
Menanga	2015	2014	Cameroon	Central	Prospective	Cross sectional	Not random selection	Urban	Hospital-based	44	13	29.5%	NR	49	NR	24	72	48	Echocardiography	Presentation with cardiac complaints + HIV
Methia	2016	2012-2013	Algeria	Northern	Retrospective	Cross sectional	Not random selection	Urban and Rural	Hospital-based	202	15	7.4%	NR	NR	NR	NR	NR	NR	Right Heart Cath	Patients with systemic sclerosis
Mocumbi	2016		Mozambique	Southern	Prospective	Cross sectional	Not random selection	Urban and Rural	Hospital-based	272	8	2.9%	NR	39	NR	NR	NR	30	Echocardiography	Presentation with cardiac complaints + HIV

Okello	2013	2010-2012	Uganda	Eastern	Prospective	Cross sectional	Not random selection	Urban and Rural	Hospital-based	309	98	31.7%	NR	NR	30	NR	NR	37	Echocardiography	Patients with rheumatic heart disease
Reuben	2015		Mauritius	Western	Retrospective	Cross sectional	Not random selection	Urban and Rural	Hospital-based	259	178	68.7%	NR	NR	NR	NR	NR	NR	Echocardiography	Patients with cardiac surgery
Sliwa	2012	2006-2008	South Africa	Southern	Prospective	Cross sectional	Random selection	Urban and Rural	Population-based	518	42	8.1%	NR	40	NR	18	72	38	Echocardiography	Presentation with cardiac complaints + HIV
Soliman	2015	2012-2013	Egypt	Northern	Prospective	Cross sectional	Not random selection	Urban and Rural	Hospital-based	51	32	62.7%	NR	59	NR	NR	NR	100	Echocardiography	Patients with chronic obstructive pulmonary disease
Stewart	2011	2006-2008	South Africa	Southern	Prospective	Cross sectional	Random selection	Urban and Rural	Population-based	5328	141	2.6%	NR	NR	NR	NR	NR	NR	Echocardiography	Patients presenting with cardiac complaints
Suiru	2015	2013-2014	Cameroon	Central	Retrospective	Cross sectional	Not random selection	Urban and Rural	Hospital-based	2194	343	15.6%	NR	NR	NR	NR	NR	NR	Echocardiography	Patients presenting with cardiac complaints
Tarrass	2006		Morocco	Northern	Prospective	Cross sectional	Not random selection	Urban and Rural	Hospital-based	86	23	26.7%	NR	45	NR	18	84	47	Echocardiography	Patients in hemodialysis
Thienemann	2016	2011-2013	Cameroon, Mozambique, Nigeria and South Africa	Multinational	Prospective	Cross sectional	Not random selection	Urban and Rural	Hospital-based	-	209	-	144 (68.9%) PH due to left-sided heart failure, 33 (15.8%) pulmonary arterial hypertension, 25 (12.0%) due to lung disease and/or hypoxia, 4 (1.9%) due to chronic thromboembolic and 33 (15.8%) with unclear/multifactorial mechanisms	NR	48	NR	NR	41	Echocardiography	Patients presenting with cardiac complaints

PH: pulmonary hypertension; NR: Not reported

* Pulmonary arterial systolic pressure > 35 mmHg for echography and mean pulmonary arterial hypertension ≥ 25 mmHg for right heart catheterization
