# Supplementary material for: Prevalence and etiologies of pulmonary hypertension in Africa: a systematic review and meta-analysis
Source: BMC Pulm Med. 2017 Dec 8;17:183. doi: 10.1186/s12890-017-0549-5 (PMC5723068; doi:10.1186/s12890-017-0549-5)
Supplement: Supplementary file 2 — Risk of bias in individual studies. (PDF 287 kb) [file 12890_2017_549_MOESM2_ESM.pdf]

Additional file 2. Risk of bias in individual studies

Studies	S1	S2	S3	C1	C2	O1	O2	O3	Score	Risk of bias
Adem, 2014	1	1	1	1	1	1	1	1	8	Low
Amadi, 2017	0	0	0	1	1	1	1	0	4	High
Amin, 2003	0	0	0	0	0	2	1	1	4	High
Amindé, 2017	1	1	1	1	1	2	1	1	9	Low
Bakari 2013	0	0	0	1	0	2	0	1	4	High
Conteh, 2016	1	1	1	1	1	2	1	1	9	Low
Ezzahra, 2015	1	0	1	1	1	2	1	1	8	Low
Ezzahra, 2015	1	0	1	1	1	1	1	1	7	Moderate
Faqih, 2016	1	0	1	1	1	2	1	1	8	Low
Gaber, 2014	1	0	0	1	0	1	0	1	4	High
Kafata, 2016	1	0	0	1	1	2	1	1	7	Moderate
Karaye, 2013	1	1	1	1	1	2	1	1	9	Low
Kingue, 2016	1	1	1	1	1	2	1	1	9	Low
Mbolla, 2016	1	0	0	1	1	2	0	1	6	Moderate
Menanga, 2015	1	1	1	1	1	2	1	1	9	Low
Methia, 2016	1	1	0	1	1	1	1	1	7	Moderate
Mocumbi, 2016	1	1	1	1	1	2	1	1	9	Low
Okello, 2013	1	1	0	1	1	2	1	1	8	Low
Reuben, 2015	0	0	0	1	0	1	1	1	4	High
Sliwa, 2012	1	1	1	1	1	2	1	1	9	Low
Soliman, 2015	1	0	0	0	0	2	0	1	4	High
Stewart, 2011	1	0	0	1	1	2	0	1	6	Moderate
Suiru, 2015	1	0	1	1	1	1	1	1	7	Moderate
Tarrass, 2006	1	0	0	1	1	2	1	1	7	Moderate
Thienemann, 2016	1	1	1	1	1	2	1	1	9	Low
